# Supplementary material for: Predicting precision grip grasp locations on three-dimensional objects
Source: PLoS Comput Biol. 2020 Aug 4;16(8):e1008081. doi: 10.1371/journal.pcbi.1008081 (PMC7428291; doi:10.1371/journal.pcbi.1008081)
Supplement: S1 Fig — Grasping patterns from human participants (left), unfitted model (middle), and fitted model (right). (a) Grasping patterns on wooden objects from Experiment 1. (b) Grasping patterns on mixed material objects from Experiment 2. (PDF) [file pcbi.1008081.s001.pdf]

Supporting Information S1 Fig for

Predicting precision grip grasp locations on three-dimensional objects

Authors:

Lina K. Klein [1,†] , Guido Maiello [1,†,*], Vivian C. Paulun [1], Roland W. Fleming [1,2]

[1] Department of Experimental Psychology, Justus Liebig University Giessen, Giessen 35394, Germany

[2] Center for Mind, Brain and Behavior, Justus Liebig University Giessen, Giessen 35394, Germany

* Corresponding Author:

Guido Maiello

Department of Experimental Psychology, Justus Liebig University Giessen, Otto-Behaghel-Str.10F, Giessen 35394, Germany

Email: guido_maiello@yahoo.it

† joint first authors; these authors contributed equally to this work

(a) Experiment 1

Human | Unfitted Model | Fitted Model

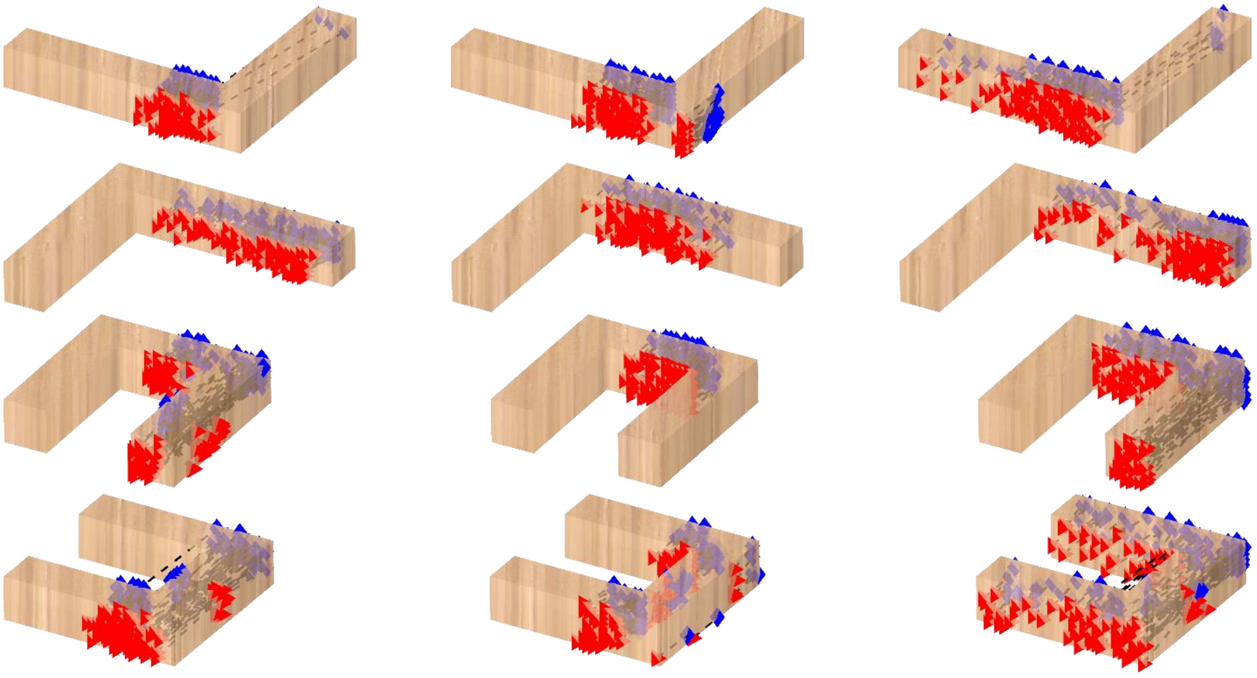

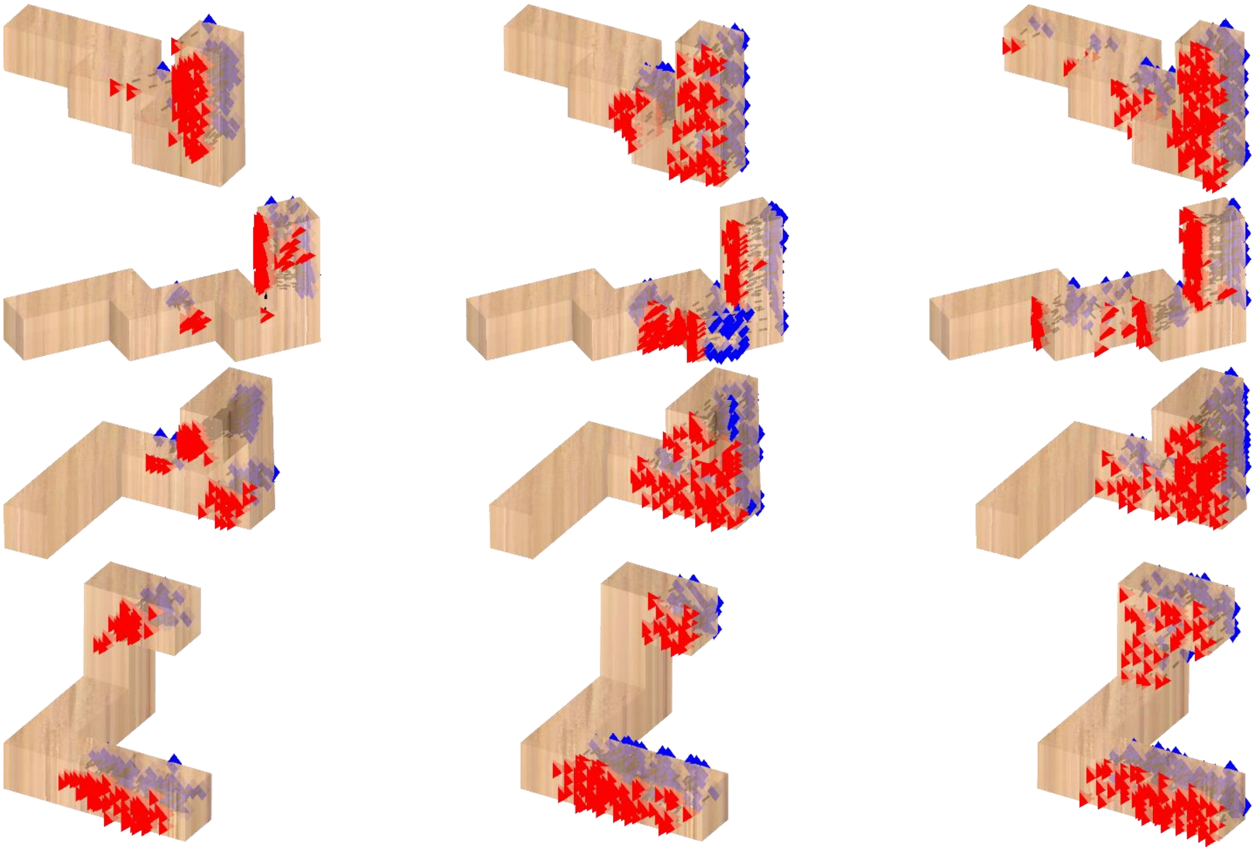

(b) Experiment 2

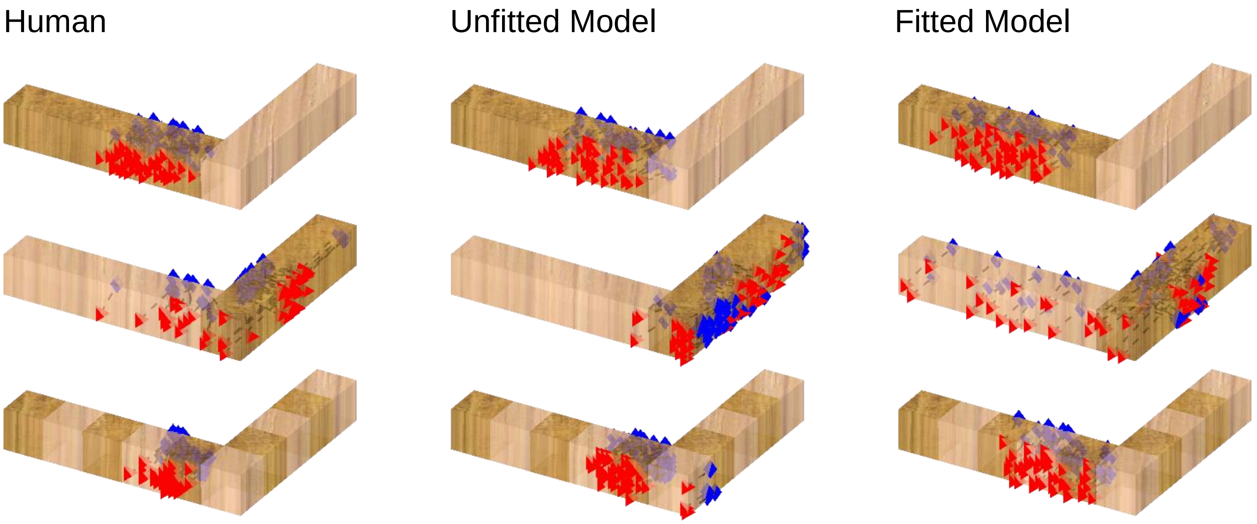

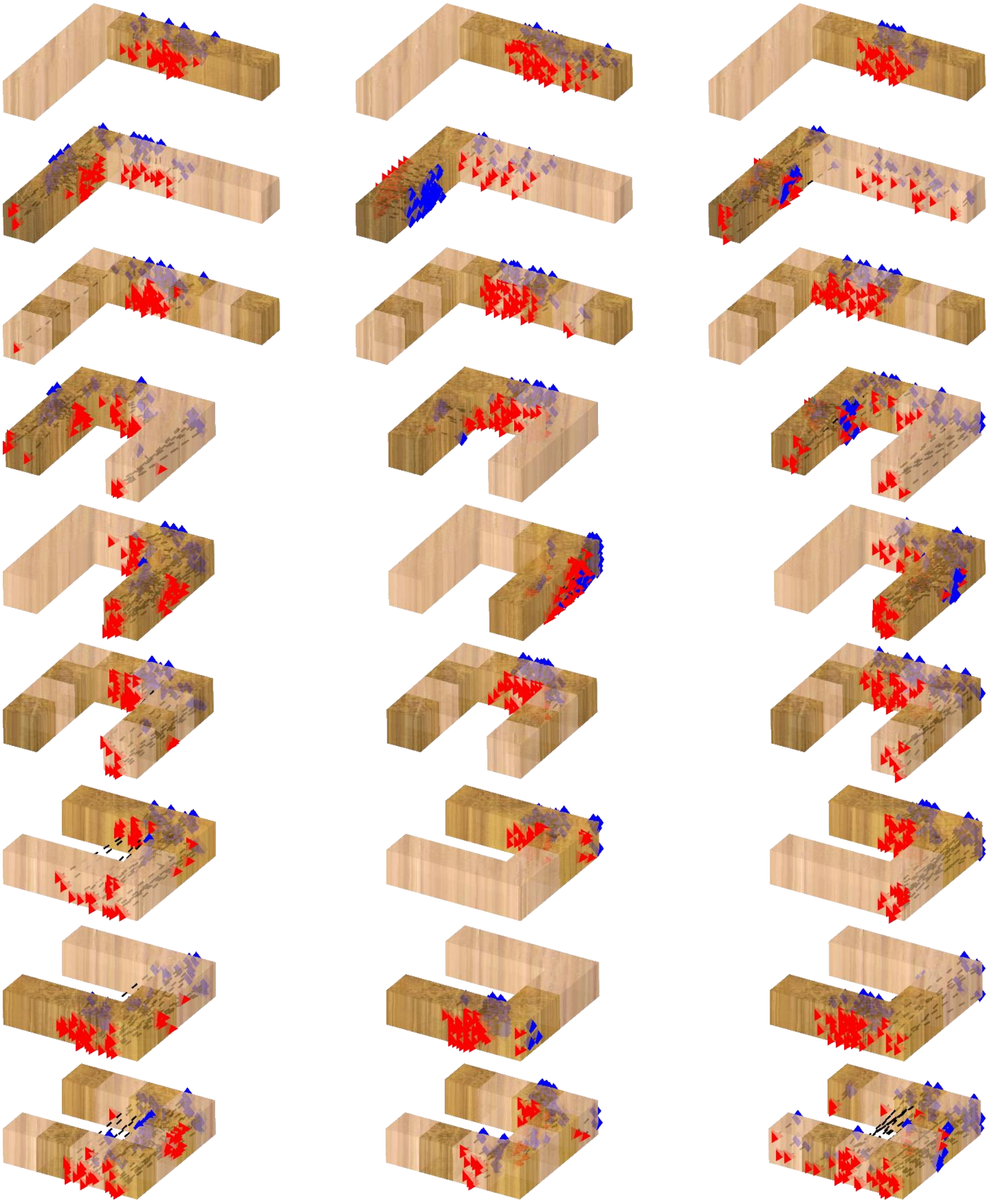

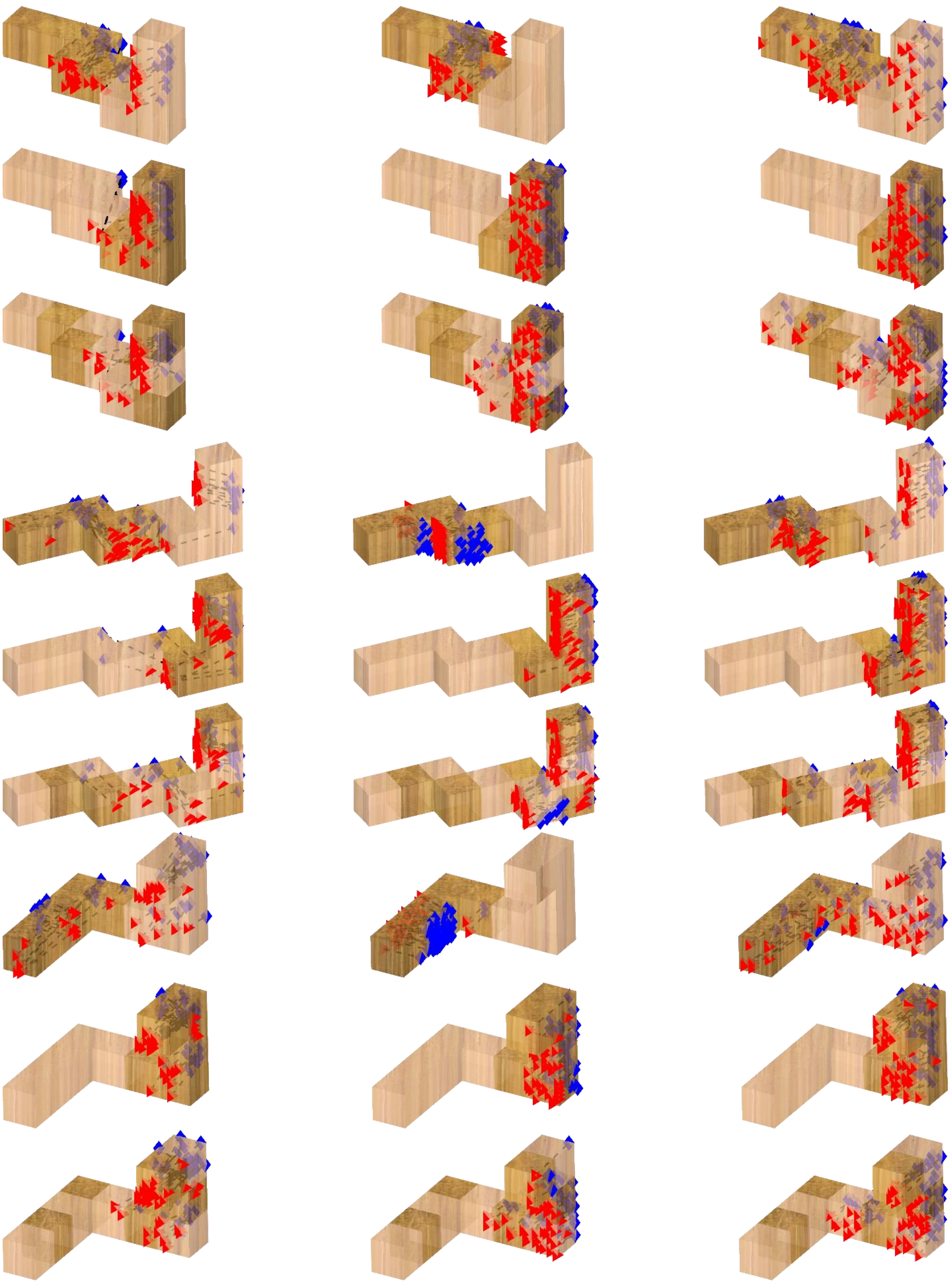

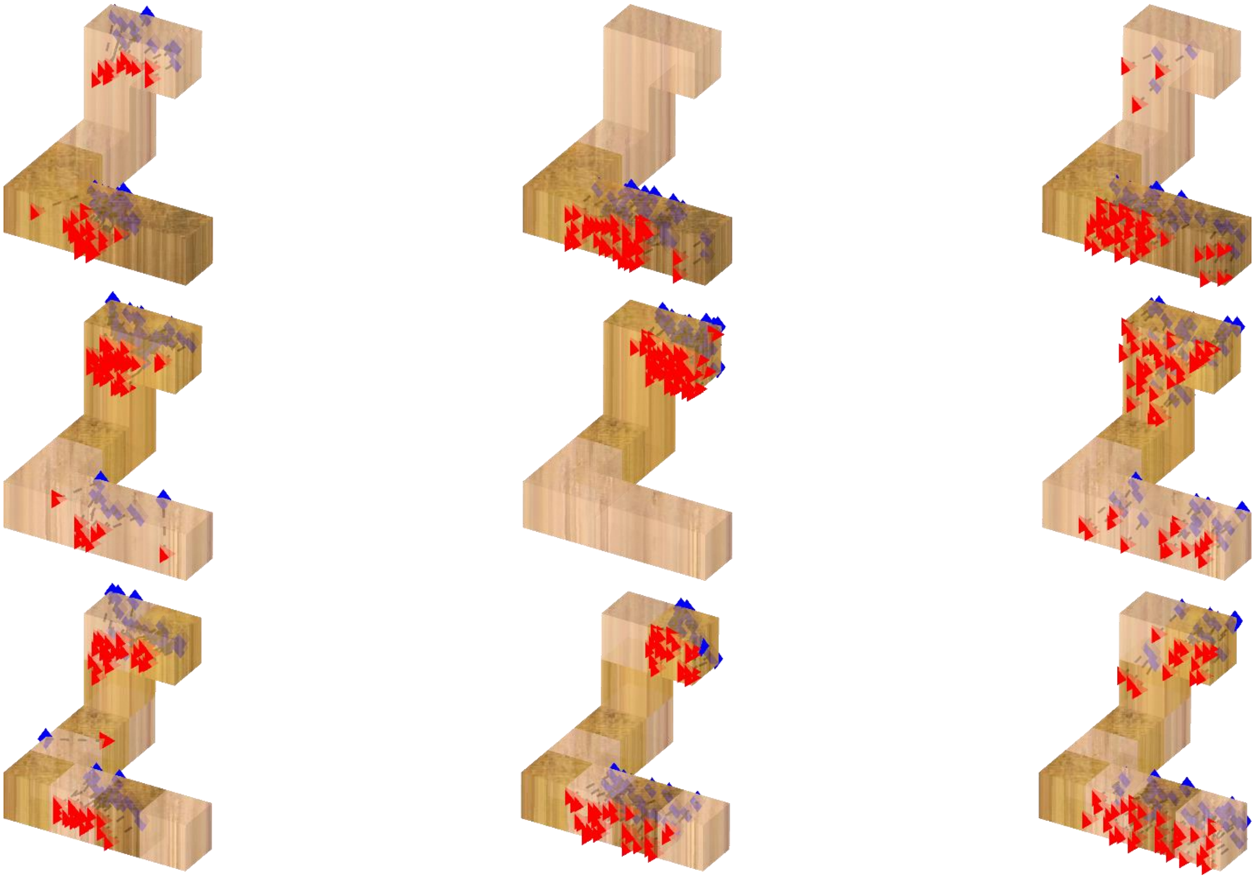

S1 Fig. Human and model grasping patterns for Experiments 1 and 2. Grasping patterns from human participants (left), unfitted model (middle), and fitted model (right). (a) Grasping patterns on wooden objects from Experiment 1. (b) Grasping patterns on mixed material objects from Experiment 2.
