# Supplementary material for: Predicting precision grip grasp locations on three-dimensional objects
Source: PLoS Comput Biol. 2020 Aug 4;16(8):e1008081. doi: 10.1371/journal.pcbi.1008081 (PMC7428291; doi:10.1371/journal.pcbi.1008081)
Supplement: S2 Fig — Panels are the same as in Figs 3, 4 and 5 of the main manuscript, except that the data are simulated from the model. The grasp trajectories in panel (4b) are from the human data, to highlight how the model correctly reproduces the biases in human grasping patterns. Panel 5b is omitted since the model cannot learn to refine CoM estimates. (PDF) [file pcbi.1008081.s002.pdf]

Supporting Information S2 Fig for

Predicting precision grip grasp locations on three-dimensional objects

Authors:

Lina K. Klein [1,†] , Guido Maiello [1,†,*], Vivian C. Paulun [1], Roland W. Fleming [1,2]

[1] Department of Experimental Psychology, Justus Liebig University Giessen, Giessen 35394, Germany

[2] Center for Mind, Brain and Behavior, Justus Liebig University Giessen, Giessen 35394, Germany

* Corresponding Author:

Guido Maiello

Department of Experimental Psychology, Justus Liebig University Giessen, Otto-Behaghel-Str.10F, Giessen 35394, Germany

Email: guido_maiello@yahoo.it

† joint first authors; these authors contributed equally to this work

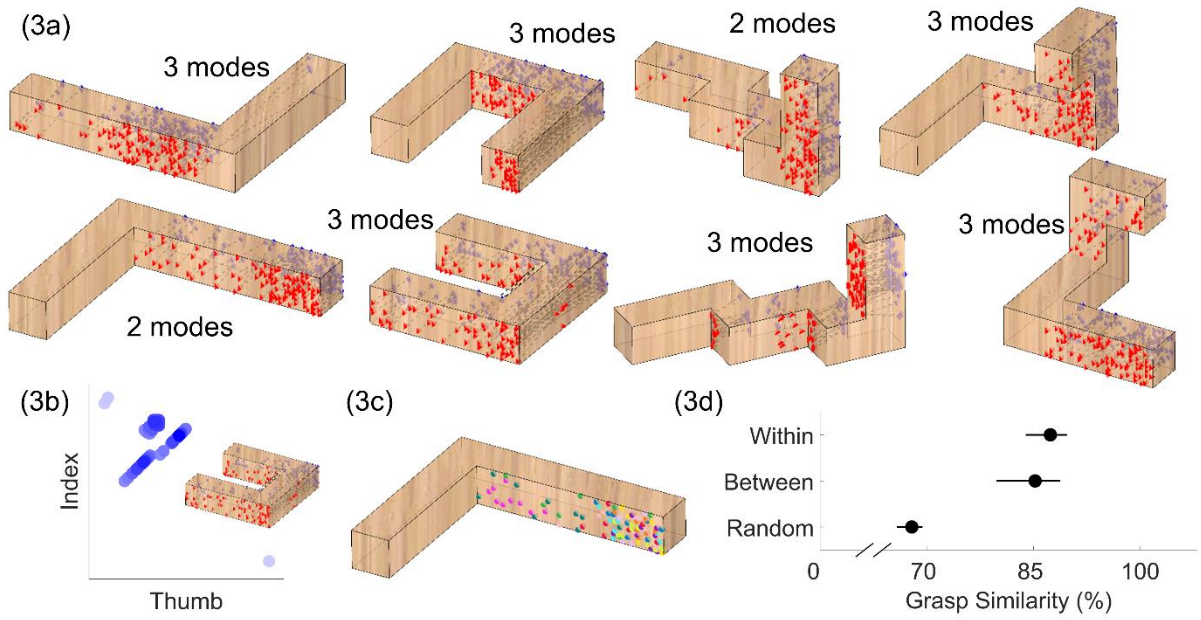

(4a)

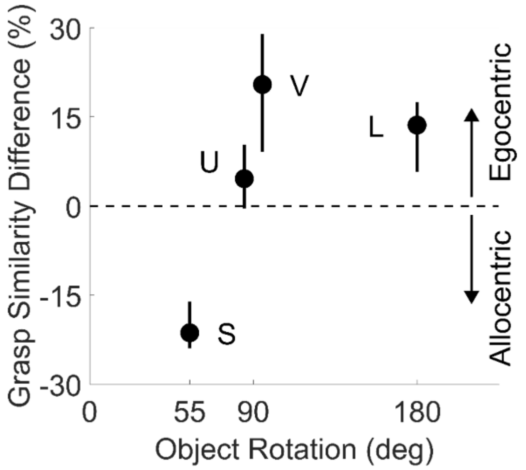

Grasp Similarity Difference (%)
30
15
0
-15
-30
V
U
L
S
Egocentric
Allocentric
0
55
90
180
Object Rotation (deg)

(4b)

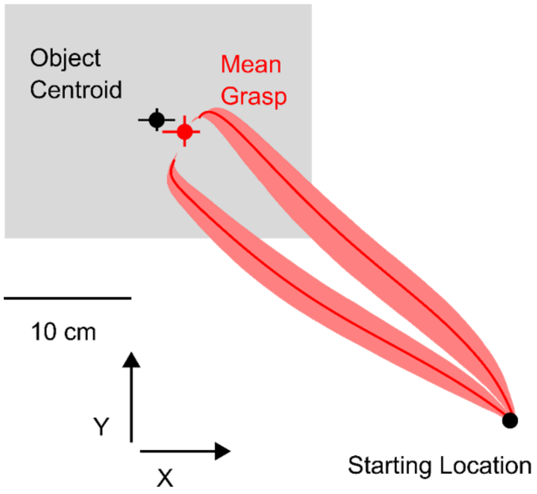

Object Centroid
Mean Grasp
10 cm
Y
X
Starting Location

(5a)

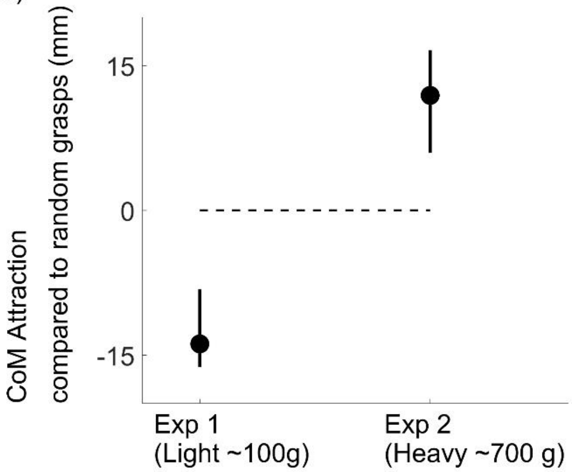

CoM Attraction compared to random grasps (mm)
15
0
-15
Exp 1 (Light ~100g)
Exp 2 (Heavy ~700 g)

(5c)

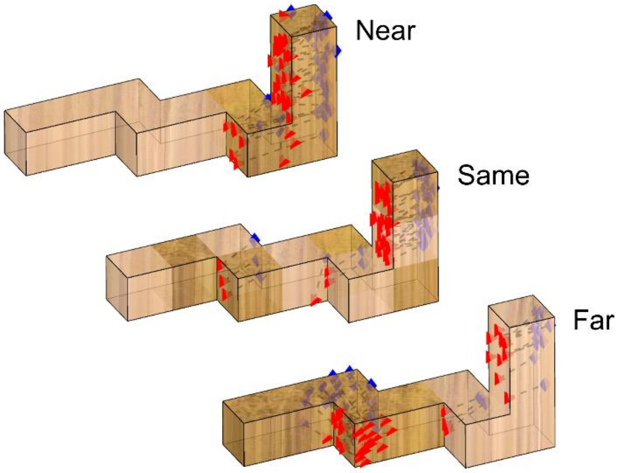

Near
Same
Far

(5d)

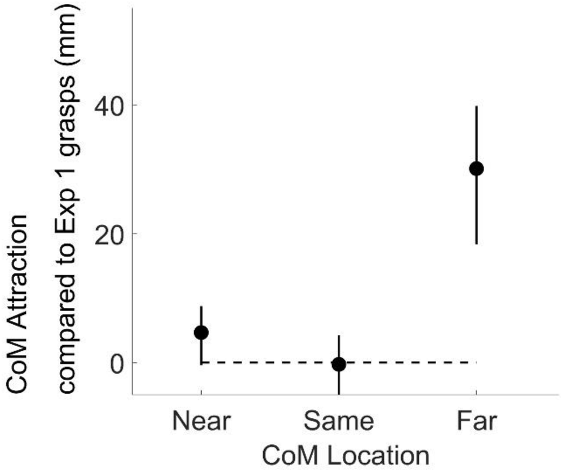

CoM Attraction compared to Exp 1 grasps (mm)
40
20
0
Near
Same
Far
CoM Location

S2 Fig. Pattern of empirical results from Experiments 1 and 2 recreated from simulating grasps from the fitted model. Panels are the same as in Figures 3, 4 and 5 of the main manuscript, except that the data are simulated from the model. The grasp trajectories in panel (4b) are from the human data, to highlight how the model correctly reproduces the biases in human grasping patterns. Panel 5b is omitted since the model cannot learn to refine CoM estimates.
