# Supplementary material for: Predicting precision grip grasp locations on three-dimensional objects
Source: PLoS Comput Biol. 2020 Aug 4;16(8):e1008081. doi: 10.1371/journal.pcbi.1008081 (PMC7428291; doi:10.1371/journal.pcbi.1008081)
Supplement: S3 Fig — The center of mass of the light wooden objects from Experiment 1 is shown as a black dot. The centers of mass for the heavy alternate and bipartite wood/brass objects from Experiment 2 are shown as red dots and squares respectively. (PDF) [file pcbi.1008081.s003.pdf]

Supporting Information S3 Fig for

Predicting precision grip grasp locations on three-dimensional objects

Authors:

Lina K. Klein [1,†] , Guido Maiello [1,†,*], Vivian C. Paulun [1], Roland W. Fleming [1,2]

[1] Department of Experimental Psychology, Justus Liebig University Giessen, Giessen 35394, Germany

[2] Center for Mind, Brain and Behavior, Justus Liebig University Giessen, Giessen 35394, Germany

* Corresponding Author:

Guido Maiello

Department of Experimental Psychology, Justus Liebig University Giessen, Otto-Behaghel-Str.10F, Giessen 35394, Germany

Email: guido_maiello@yahoo.it

† joint first authors; these authors contributed equally to this work

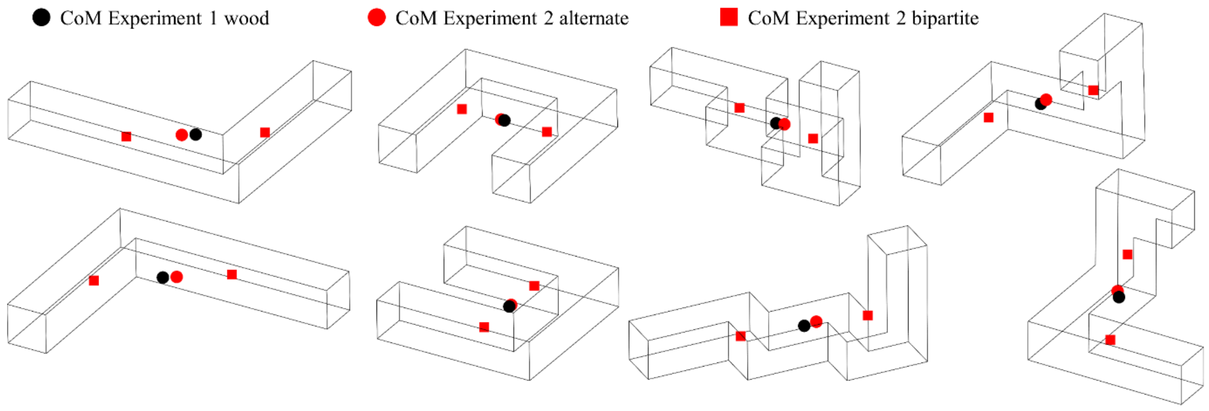

S3 Fig. Location of the center of mass for the stimuli employed in Experiments 1 and 2. The center of mass of the light wooden objects from Experiment 1 is shown as a black dot. The centers of mass for the heavy alternate and bipartite wood/brass objects from Experiment 2 are shown as red dots and squares respectively.
