# Supplementary material for: Predicting precision grip grasp locations on three-dimensional objects
Source: PLoS Comput Biol. 2020 Aug 4;16(8):e1008081. doi: 10.1371/journal.pcbi.1008081 (PMC7428291; doi:10.1371/journal.pcbi.1008081)
Supplement: Attachment — Submitted filename: Response_to_reviewers.pdf [file pcbi.1008081.s004.pdf]

We thank all three reviewers for the positive assessment of our work and for the constructive feedback we address below.
Reviewer comments are written in italics. Our responses are in bold and inset. Changes to wording in the manuscript are highlighted in red text. Line and page numbers refer to the revised manuscript with tracked changes.

Reviewer #1: The manuscript addresses an important topic and it provides an interesting development of the current views about how people select the contact points when they have to grasp objects. The authors acknowledge that this is an initial attempt at addressing this topic and it thus doesn't capture yet the full spectrum of possible grasping actions (e.g., multi-digit grasping, grasping complex shapes with curved surfaces, etc.). Nevertheless, it is a good contribution to this topic. I also had the chance to read the opinions of the previous reviewers who raised a number of important points which were successfully addressed by the authors.

Here below I'll list some elements that I think still need to be addressed.

line 254, Optimal grasp aperture: the authors say that 2.5 cm is the preferred distance between finger and thumb for precision grips citing Cesari and Newell, but in the present case the participants were explicitly instructed to use a precision grip, so I don't see why the 2.5 cm were chosen as an important value to define this penalty function. It would have made more sense to measure the maximum comfortable grasp aperture span of each participant and use those values to define the range of possible grasp apertures. Any grasp aperture between 0 and the maximum comfortable grasp aperture span should have a penalty function of 0. How robust is the computational model to different versions of the optimal grasp aperture penalty function such as the one I have mentioned above?

> **We thank the reviewer for this observation. In Cesari and Newell, cubes of 2.54 cm were exactly where participants switched from using two digits to using three. Therefore, we argue that for precision grip participants should avoid grip apertures larger than this threshold, since for these grip apertures participants should prefer multi-digit grasps. In pilot work, we found that the specific threshold had little influence on model performance, and even a penalty function linearly increasing from 0 worked well. We've now further run the perturbation analyses suggested by the reviewer and we've included these in the revised manuscript (page 24 line 453):**
>
> "Since the backside of objects is occluded from view, it is unlikely that participants have an accurate estimate of the required grip aperture across the whole object. Additionally, since we constrained participants to two-digit precision grips, grasps above the threshold defined by Cesari and Newell (26) might be acceptable, as long as these are within a maximum comfortable grasp span. Figure 14b shows that indeed model performance is robust to increases in aperture threshold up to 100 mm."
>
> **As suggested by the reviewer, only when employing a threshold that approaches a maximum comfortable grasp aperture span does model performance decrease. However, keeping the current threshold will allow us, in future work, to extend our current modelling to multi-digit grasps by employing multiple thresholds between**

different multi-digit grasps, as found in Cesari and Newell. We now include these considerations in the discussion (page 29 line 566):

"The model should also be extended to multi-digit grasping, by adding to each penalty function three dimensions for each additional finger considered (the x,y,z coordinates of the contact point). This approach is consistent with (and complementary to) the approach by Smeets and Brenner (2, 5), who posit that grasping is a combination of multiple pointing movements. Given that human participants adjust the number of digits they employ to grasp an object depending on grip size and object weight (26), multiple size/weight thresholds could be employed to determine the preferred multi-digit grip."

And methods sections of the revised manuscript (page 40 line 828):

"In pilot work, we observed that a penalty map linearly increasing from 0 cm worked equally as well as one linearly increasing from 2.5 cm. In Experiment 3 we further observed that increasing this threshold up to 10 cm did not hinder model performance. However, constructing this penalty function with the 2.5 cm threshold motivated by previous literature will allow us, in future work, to construct penalty functions with multiple thresholds for multi-digit grasping, as those observed by Cesari and Newell (26)."

One aspect that is not mentioned in the manuscript is the fact that participants might have actually learned about the properties of the object during an experimental session. Did contact point selection change over the duration of an experimental session when participants had the chance to interact multiple times with the various objects and thus learn the characteristics of each object? For example, are the contact point locations more clustered if you compare the last trials with the first trials?

We thank the reviewer for this interesting question. We've performed the suggested analysis and found that participants did not adjust their strategy in Experiment 1, but did refine their preference for grasps close to the CoM in Experiment 2. We now include these observations in the revised manuscript for Experiment 1 (page 7 line 129):

"We observe no obvious learning effects across trial repetitions: between-subject grasp similarity does not change from first to last repetition across objects and orientations ($t(7)=0.62$, $p=0.56$)."

And for Experiment 2 (page 12 line 226):

"Figure 5b shows how this behavior was evident already from the very first trial performed by participants, but also that grasps clustered more toward the object CoM in later trials, presumably as participants refined their estimates of CoM location (correlation between CoM attraction and trial repetition: $r = 0.86$, $p = 0.13$)."

Line 215: The authors say that they do not suggest that the human brain explicitly evaluates grasp cost for all possible locations. This makes a lot of sense. However, another aspect that is also not very realistic is that humans actually have a VERIDICAL representation of the 3D object shape, its mass distribution, orientation and position that would be needed to even start any process similar to the one suggested by the authors. It would be important to mention also this aspect and it would be interesting to implement some perturbations to the inputs of the computation model and observe how robust the model is to them. For example, the 3D description of the shape could be slightly distorted, the mass distribution could not be perfectly accurate, the orientation and position slightly off: if the model is then still able to decently match human performance it would add value to the suggested model.

We agree with the reviewer that testing whether the model is robust to perturbations would strengthen the study. Therefore, in response to this and to the reviewer's first comment, we've added a section of the manuscript investigating model performance with different kinds of perturbations (page 24 line 441):

"**Model Perturbation Analysis.** The model designed thus far receives as input a near-veridical representation of the objects to grasp. However, it is unlikely that humans have access to such a veridical object representation. We therefore implemented some perturbations to the inputs and key parameters of the computation model and observed how robust the model is to these perturbations. Specifically, we tested how model performance in predicting human grasping patterns from Experiment 3 varies as a functions of these perturbations.

The model input thus far consisted of densely sampled 3D mesh models. It's unlikely that humans also have such a dense, accurate 3D representation of an object's surface. Figure 14a therefore shows model performance (in terms of similarity with human grasping patterns) with different levels of surface mesh subsampling. Model performance is robust to relatively high levels of subsampling, and decreases only once sampled surface locations are on average more than 4 mm distant from one another (beyond 5% mesh subsampling).

Since the backside of objects is occluded from view, it is unlikely that participants have an accurate estimate of grip aperture across the whole object. Additionally, since we constrained participants to two-digit precision grips, grasps above the threshold defined by Cesari and Newell (26) might be acceptable, as long as these are within a maximum comfortable grasp span. Figure 14b shows that indeed model performance is robust to increases in aperture threshold up to 100 mm.

Similarly, humans might also exhibit some tolerance for grasps oriented away from the natural grasp axis. Given that the ease of a rotation of the arm and hand is likely asymmetric along different directions, these tolerances likely also vary depending on rotation direction. Figure 14c shows how model performance does indeed decrease for perturbations of the natural grip axis along the transverse plane, and this decrease is more steep for clockwise (negative) rotations, as already suggested by Kleinholdermann and colleagues(15). Model performance is instead more robust to perturbations along the sagittal plane (Figure 14d), and particularly for (positive) counterclockwise rotations in which the thumb tilts below the index finger."

Figure S2: I would like the authors to speculate also about some surprisingly bad predictions of their model. For example, in Figure S2 (a), line 4, the Fitted Model predicts some grasps which are opposite to those in Human (thumb is predicted to land where humans placed the index, and the index is predicted to land where the humans placed the thumb). Or, in line 6, there is a cluster of index contact points on the lower right side that doesn't exist in human data and it seems to lack its corresponding thumb contact points (or are the predicted grasps here actually on the two sides of the corner?).

Thanks to the reviewer's comment, we've realized that this figure had not been correctly updated from the previous iteration of the model which had a different model fitting procedure. We've now updated this figure and double checked that all other figures and results are up to date. In this updated figure, most of the "surprisingly bad predictions" pointed out by the reviewer are no longer present. There are a handful of model grasps in which finger and thumb are switched, which is most likely due to the model equally penalizing clockwise and counterclockwise azimuth rotations away from the NGA, whereas human participants likely prefer counterclockwise rotations. We now note this in our new perturbation analyses (page 24 line 462):

"Figure 14c shows how model performance does indeed decrease for perturbations of the natural grip axis along the transverse plane, and this decrease is more steep for clockwise (negative) rotations, as already suggested by Kleinholdermann and colleagues(15)."

Typos:
line 215, typo: "are be optimal"

Thanks, changed to: "are ~~be~~ optimal"

line 216, unclear: "It provides a means of based on a subset..."

Thanks, changed to: "It provides a single, unifying framework ~~a means of~~ based on a subset of the factors that are known to influence human grasp selection ~~into a single, unifying framework~~"

line 454: though should be through

Thanks, fixed.

Figure S3, (a): "2 mode" should be "2 modes".

Thanks, fixed

Reviewer #2: This manuscript describes sets of human-subjects experiments that investigate how people visually plan and grasp six differently shaped and oriented objects, using line of sight observation tracking to determine the positioning of the index finger and thumb. Based on this data, the results indicate high similarly between subjects, within an object. Based on the tracking data, a data-driven model involving several parameters is built to assess the relative importance of each (force closure, torque, axis, etc.) on grasp selection.

Overall, the experiments are well planned and conducted, and their resultant data are rigorously analyzed. The question of the visual planning of initial grasping location based on visual cues of an objects geometry, orientation, etc. is an interesting one, and builds upon prior work in this area. A model that seeks to unify the data from various studies could be a nice addition to the literature.

Major comments:
****
1. The experiments do leave one with a clear sense that particular grasp elements are more important than others. On the other hand, while the model seems to be the major contribution of this work, it is preliminary and not well enough articulated. First, the model is built up from observations with a set of objects and then used with those same set of objects, so one wonders how well is applies to a new set of objects or new case. Therefore, it seems like it well describes the observed phenomena from the human subjects experiments, but it's not clear how predictive it is of new situations/objects. This is problematic because most of the introduction and other sections denote it's predictive ability. Second, even within what it does, I find its articulation (mostly done through Figure 4) hard to follow, and wonder if the authors could focus more on a single concrete example, of greater detail. One of the selling points of the MS is that there is a unified model that brings together findings from theirs and other studies, but if this is the central contribution, more time, space and figures should be spent on it, and demonstrating it. And I also wonder if the authors should generate new objects to determine how well it predicts grasp for those.

We agree with the reviewer that to substantiate our claim that the model is predictive we must test it on novel objects. We have added a section to the manuscript where we show model predictions on novel objects and find that these predictions indeed align with human grasps (page 23 line 420):

"**Model Predictions on Novel Objects.** The model was designed from the insights derived from Experiments 1 and 2 with polycube objects made of brass and wood. To test whether the model generalizes beyond this type of object, we selected four mesh models of objects with smooth, curved surfaces from an in-house database. We input these meshes to the model and generated grasp predictions (Figure 13a). The model was instantiated using the weights derived from Experiment 1. Next, we 3D printed these objects out of light plastic (~80g, comparable to Experiment 1 objects), and asked 14 human participants to grasp these novel objects. Figure 13b shows how human grasps agree with model predictions. Human and model grasps once again have similar size and orientation, and also cover similar portions of the objects. Figure 13c confirms this

observation: predicted model grasps are as similar to medoid human grasps as grasps from a random human participant (t(13)=1.53, p=0.15)."

To strengthen the description of the model, we now include several new figures (Figures 7, 8, 9, 10, 11) showing examples of how the model is constructed.

2. The current introduction is not well motivating the study. First, it's not precise in several regards surrounding "computation," as well as in whether grasp selection is initial selection or later selection and if feed-forward information can be employed because the object has now been touched several times, etc. Once you get to the section in the lines 50-65 range, you figure out where the MS is positioning itself, but before that point, I'm wondering where this manuscript is headed. The language and writing is quite loose. Second, all through reading that part, I'm asking myself these questions because of my familiarity with seminal work by Roland Johansson, and I'm wondering when I will hear about their work relative to it. The authors bring up Johansson, but not until the Discussion. After reading the whole MS, I do recognize what they're doing is different. However, because that Johansson work is so prominent, the introduction must address it.

We acknowledge that "computation" means different things to different readers, leading to uncertainty. To address this, we have (1) changed the term "computational model" to "normative model" at many locations in the manuscript, (2) now already state explicitly what the input and output of the model are in the introduction (page 5 line 77):

"The model takes as input a near-veridical 3D mesh representation of on object to be grasped, performs free-body computations on the mesh, and outputs minimum-cost, optimal grasp locations on the object."

and (3) better specify that we consider initial grasp selection (page 4 line 41):

"…little is understood about the computational basis of initial grasp selection"

and (page 4 line 61):

"We measured initial contact locations (i.e. not readjusted contact regions during movement execution)."

As we understand it, the work by Johansson predominantly deals with grasp stability: how the sensorimotor system adjusts grasps based on tactile afferent signals and how object properties, such as slipperiness of the object's surfaces, affect grip force. We now cite this work in the introduction of our manuscript: (page 4 line 40):

"Despite the extensive literature describing human grasping patterns, ~~and~~ movement kinematics, and grip force adjustments (2–14)~~(2–11),~~…"

However, while Johansson's work is indeed important and well-known, it does not directly relate to our study. We only investigate initial contact with the object (i.e. grasps are not adjusted) and we do not currently measure grip forces nor the objects' surface friction. Therefore, we do not feel it is appropriate to extensively discuss Johansson's work in the Introduction of our manuscript. As noted by the reviewer we do of course discuss Johansson's work in the Discussion section of our manuscript.

3. Several key terms are either unclearly defined or misused. The authors use the term "computational model" in many places, and many readers (including me) would might say this is not a computational model. It is essentially a data-driven, regression-like model, that is a not a physics based, but is built up from observations of people interacting with objects. They also use the term "predict" but they do not per se predict how well the model can be applied in using new objects, as previously noted. It works well with objects it was built around, and perhaps it could do well for new objects, but that's not shown. Later in the manuscript, the term "normative" model is used. The authors need to fix the "computational" and "predict" terminology to be accurate with what was done. Another term used in the abstract and various other places regards material properties, but the objects used in these studies rigid bodies with flat, smooth surfaces, so the material properties are not varied. It could be however, that the wood and brass do present different coefficients of friction, which might be problematic. Finally, it would be good if the authors were more clear that they are looking at the "initial grasp position" rather than the "readjusted during the grasp position". And their force closure is more simplistic than the grip and lift forces that Johansson describes.

We acknowledge that the term "computational model" might have a slightly different meaning in different subfields. To avoid confusion, we now refer to the model as a "normative model" at most places in the manuscript, including in the abstract and the heading of the section that described the model. Given that computational operations are performed on digital 3D objects to produce the output (model grasp locations on the object), we feel that the term 'computational' to describe the model is appropriate, so we retained it in some locations. We disagree that the model is regression-like. The unfitted model is precisely that: it has zero free parameters, does not take human data as input and still well accounts for the human data. Thus the unfitted model's outputs are formally a prediction, not a description, of the human data. Even when we fit the model we do not perform classical regression procedures as we had to develop a fairly complex fitting procedure given the nature of the input 3D meshes and the relatively sparse human data. In our view the model is a physics-based, normative model, and we now better specify this throughout the manuscript. We also now test model predictions for novel objects, as suggested by the reviewer and discussed above.
Wood and brass are indeed different materials, and a key aspect is that they have different density and therefore different mass, a key component of the study that warrants the mention of material properties. We are however now more careful about referring to the specific material property we vary.
While we were unable to find measurements of friction coefficients for beech wood and brass against skin, our reading of the literature suggests that these should be

comparable but nontrivial to measure. We now comment on this point in the revised manuscript (page 28 line 554):

"Incorporating friction into the model could even improve model performance for our composite objects from Experiment 2, as wood and brass may have different friction coefficients. Since surface friction plays a decisive role in determining force closure, friction coefficients could even be directly integrated into the force closure computations. Friction is also a particularly interesting test case for our assumption of a weighted linear combination of costs, as it may interact with other factors. When friction is low, it could cause the cost of torque to be upregulated, to avoid slipping. This would require the addition of parameters describing interactions between factors. Alternatively, friction and torque might be unified into a single penalty function capturing the magnitude of grip force required to avoid slippage. However, incorporating friction into the model would be non-trivial, since the coefficient of friction between skin and different materials depends on several factors, including temperature, hydration, and age (44)."

As mentioned above, we now better specify that we consider initial grasp selection. Also note that grip and lift forces do not influence force closure computations. We now specify this in the revised manuscript (page 38 line 796):

"A grasp that does not fulfill force closure will not be able to lift and freely manipulate the object, no matter the amount of force applied at the fingertips."

4. Like Fig. 4, but more to a point of formatting, I think Fig. 3 (described below) should be re-formatted or broken up into distinct figures. At present, there are too many sub-figures, with too small of text, of unclear relationship to one another.

We appreciate the suggestion and have split the figure in three distinct figures:

"**Figure 3.** Human grasps are clustered. ~~Empirical Results~~ (a) Human grasps from Experiment 1. Grasps are represented as thumb (red triangles) and index finger (blue diamonds) contact positions, connected by dotted black lines. (b) Human grasps (blue blobs) for object U, orientation 2, when projected in a unitless 2D representation of the space of potential grasps, cluster around three distinct modes. (c) Distribution of thumb contact points on object L, orientation 2. Different colors represent grasps from different participants. (d) The level (%) of grasp similarity expected for grasps randomly distributed on the object surface (i.e. random combinations of thumb and index finger positioning attemptable on an object) and the observed level of between- and within-participant grasp similarity, averaged across objects and orientations. Error bars~~/regions represent~~ are 95% bootstrapped confidence intervals of the mean. ** $p<0.01$, *** $p<0.001$~~Difference in grasp similarity across orientations when grasps were encoded in object-centered (allocentric) vs human-centered (egocentric) coordinates, as a function of magnitude of rotation across the two orientation conditions. (f) Average grasp trajectories viewed in the x-y plane (red curves) from start location towards the objects (always contained within the gray shaded region). The average human grasp (red dot) across conditions is biased toward shorter reaching movements compared to the object centroids~~

(black dot). (g) Attraction towards the object CoM for grasps executed onto light (Experiment 1) and heavy (Experiment 2) objects compared to the average CoM distance of grasps uniformly distributed on the object surfaces (zero reference). (h) Human grasps from Experiment 2 onto object S presented at orientation 2. (i) Attraction towards the object CoM compared to Experiment 1 grasps (zero reference), for Experiment 2 grasps onto heavy objects whose CoM is closer, the same distance as, or farther than the light wooden objects from Experiment 1. In all panels, e"

"**Figure 4.** Spatial encoding and bias. (a) Difference in grasp similarity across orientations when grasps were encoded in object-centered (allocentric) vs human-centered (egocentric) coordinates, as a function of magnitude of rotation across the two orientation conditions. (b) Average grasp trajectories viewed in the x-y plane (red curves) from start location towards the objects (always contained within the gray shaded region). The average human grasp (red dot) across conditions is biased toward shorter reaching movements compared to the object centroids (black dot). In both panels data are means, error bars/regions represent 95% bootstrapped confidence intervals. *** $p<0.001$"

"Figure 5. Mass and Mass Distribution. (a) Attraction towards the object CoM for grasps executed onto light (Experiment 1) and heavy (Experiment 2) objects compared to the average CoM distance of grasps uniformly distributed on the object surfaces (zero reference). (b) Attraction towards the object CoM in Experiment 2 as a function of trial repetition. Red line is the best-fitting regression line through the data (c) Human grasps from Experiment 2 onto object S presented at orientation 2. (d) Attraction towards the object CoM compared to Experiment 1 grasps (zero reference), for Experiment 2 grasps onto heavy objects whose CoM is closer, the same distance as, or farther than the light wooden objects from Experiment 1. In panels a,b, and d, data are means, error bars represent 95% bootstrapped confidence intervals. ** $p<0.01$, *** $p<0.001$"

Detailed comments:
****
A. Abstract, Last sentence: "Together, the findings provide a unified account of how we successfully grasp objects with different 3D shapes and material properties." - The authors mention material properties. The other factors mentioned are shape, weight, orientation, and mass distribution but these are not material properties, per se as one would view from a mechanical engineering perspective. Material properties are elasticity, and/or it's time dependent nature, hysteresis, and surface texture, etc. This should be clarified.

We've changed the text to (page 2 line 14):

"Together, the findings provide a unified account of how we successfully grasp objects with of different 3D shapes, orientation, mass, and mass distributionterial properties."

B. Intro, about lines 36-48. Moreover, Westling and Johansson, 1984, "Roles of glabrous skin receptors and sensorimotor memory in automatic control of precision grip when lifting rougher or more slippery objects" have done extensive work on grasp, and looked at the ratio of lift to grip forces in picking up various objects and how people use safety factors to ensure the object

does not slip on them, they also considered the use of feed-forward visual estimation that drives selection of the appropriate grasp, and in their experiments varied the surface contact interactions by using various materials, from sand paper to slippery velvet, etc. This paper and other papers of theirs have been cited thousands of times, and are of mainstream thought in the field of haptics. The MS does cite 3 Johansson papers but only in the discussion section.

As mentioned above, we have updated the introduction to include this and other references to Johansson's work (page 4 line 40):

"Despite the extensive literature describing human grasping patterns, ~~and~~ movement kinematics, and grip force adjustments (2–14)~~(2–11),~~…"

C. Intro, Line 22, "computational complexity" seems to refer to how humans perform the task, or is it how AI performs the task? It should be clarified as to if the authors are implying that humans are using computational processes to make decisions about grasp and lift tasks, or if they are referring to sensors and actuators on robots and the associated computational and/or analytical algorithms used to inform grip adjustment. This terminology is also used in Figure 1's caption and elsewhere.

We are drawing a parallel between the computations performed by humans and those performed by robotic AIs. We've reworded the text for clarity (page 3 line 25):

"However, this ease belies ~~it's~~ the computational complexity of human grasping."

And (page 3 line 37):

"Figure 1. The computational complexity of human grasp selection."

As mentioned above, for clarity we have changed the term "computational model" to "normative model" throughout the manuscript.

D. Intro, lines 49-50, "we sought to unify these varied and fragmented findings into a single computational framework." This is not really a hypothesis, per se. What comes after this mentions constructing a dataset and teaching apart the five factors. And then onto the factors varied per experiment, but this writing is fairly loose and not tied tightly enough to a precise goal and to the prior literature. After reading this section, I'm struggling to determine the direction of this work, especially without any tie to the seminal Johansson references.

Our preference in writing style is to begin with a broad introduction to the topic and progressively narrow down to the specifics of the current work. In our view, this favors comprehension for readers who are not intimately familiar with the topic. Extensively discussing Johansson's work on grip forces in the introduction, when our work does not directly address this topic (as also the reviewer points out), would likely confuse non-expert readers. We have nevertheless updated the introduction

following several of the reviewer's suggestions, please see our responses to previous comments.

E. Intro, lines around 45, In this paragraph, sometimes the language is from the object's perspective (e.g., mass, mass distrib, shape) and others from the person's grasp (orientation, size). Make it consistent for the case being described.

Fixed: (page 4 line 47):

"Object ~~Mass~~ mass and mass distribution must be evaluated, since for grips with high torques (i.e. far from the center of mass, CoM(18–22)~~(15–19)~~) the object will tend to rotate under gravity and potentially slip out of our grasp (Figure 1c, top). The orientation(19, 23–25)~~(16, 20–22)~~ and size(26)~~(23)~~ of grasps on an object must be considered, since the arm and hand can move and apply forces only in specific ways."

F. Intro, lines around 45, In this paragraph, since CoM may be hard to interpret visually for some strange geometrics, are we just restricting the grasp to the initial grasp? Or readjusted in mid-lift? Also, the second time the participants have seen it, they would have some feed-forward information. Would they have adjusted their grasp the second time they saw a configuration? Was this last point analyzed?

We now better specify in the text that we are analyzing initial contact points, not readjusted grasp locations (page 4 line 61):

"We measured initial contact locations (i.e. not readjusted contact regions during movement execution)."

We have further included an additional analysis, as suggested also by Reviewer 1, in which we show that participants did not adjust their strategy in Experiment 1, but did refine their preference for grasps close to the CoM in Experiment 2. We now include these observations in the revised manuscript for Experiment 1 (page 7 line 129):

"We observe no obvious learning effects across trial repetitions: between-subject grasp similarity does not change from first to last repetition across objects and orientations ($t(7)=0.62$, $p=0.56$)."

And for Experiment 2 (page 12 line 226):

"Figure 5b shows how this behavior was evident already from the very first trial performed by participants, but also that grasps clustered more toward the object CoM in later trials, presumably as participants refined their estimates of CoM location (correlation between CoM attraction and trial repetition: $r = 0.86$, $p = 0.13$)."

G. It is very difficult in figure 3 to make sense of how all of these figures tie together. Some have aggregate data for all participants, some appear to be for one or another

factor or case, etc. What is the story to be told by these? Figure 3 should be reformatted or broken into more than one figure. Figure, 3b - what are the axis units and labels? Figure 3f - How does this figure tie to the others? And is this just an example figure? How is the 26 mm noted to be gleaned from Figure 3f? The figure is quite small and it is unclear if the axes represent quantities to scale.

As mentioned above, we appreciate the criticisms and have split the figure in three distinct figures for clarity.
In Figure 3b we now specify that the axes are unitless, which is why we omit them. Figure 3f is not an example, we would specify this if it were the case. As specified in the figure legend, these are averaged grasp trajectories. We do not expect readers to eyeball the 26mm from the figure, which is why we specify it in the text. However, distances are indeed represented to scale, as indicated by the scale present in the panel.

H. Intro, line 65, a model is mentioned. At this point, the MS needs to be more specific on what are the input-output relationships and what type of model (regression, free-body, dynamics, solid mechanics, etc.). I think it would also be good to be clear that w.r.t. force closure and torque that these are acquired by free-body physics, and involve no direct measurements of force related quantities which might come from sensors on the device itself. I'm not sure how you can get actual torque if you do not have normal force and are not tracking the movement of the block pattern.

We clarified the nature of the model as suggested (page 5 line 77):

"The model takes as input a near-veridical 3D mesh representation of on object to be grasped, performs free-body computations on the mesh, and outputs minimum-cost, optimal grasp locations on the object."

We specify in the methods that to estimate torque we make the assumption that is possible to apply forces at the thumb and index contact points that counteract the force of gravity, making it possible to derive a computable equation for torque.

J. Results, line 156-7. "...our findings suggest humans care little about torque when grasping lightweight objects." This statement should be quantified, if left in results, or else moved to methods if meant to be brought up in a higher-level, abstract way. What torque range are we referring? What range of weights of objects are we referring?

We have included this information (page 10 line 188):

"lightweight objects (of ~100 g)."

K. Results, in several points between lines 160-195. It seems like the participants are estimating CoM by their own volition. They were not taught how to do this, but perhaps they make guesses in their head as to the configurations of the wood and brass blocks, and resulting CoM? Did you consider painting these so that participants would not know the composition of block patterns

that they're pickup up? Perhaps they participants when doing this task see the metals, think they might be heavier regardless of configuration, and therefore they choose a safer position, rather than faster/easier in the all wood case.

The participants knew the relative weight of brass and wood, as they were familiarized with these materials using test objects, which we specify in the methods section of the manuscript. Participants were likely able to estimate CoM locations for the composite objects since recent work from co-author Paulun has shown that participants are fairly accurate at pointing at the CoM of bipartite objects made of two different materials. We now cite this work in the revised manuscript (page 27 line 520):

"More recent work by Paulun et al. has further shown that participants are fairly accurate at visually judging the location of the CoM even for bipartite objects made of two different materials (39)."

We agree with the reviewer that it would be interesting to test whether participants are able to accurately estimate the CoM of objects of complex shape and made of multiple materials, but this was not the purpose of the current study.

L. Results, lines 241-265. This section discusses the metrics and penalty function calculations. Instead of talking about these in a general way, why not tie these directly to Figure 4. The rest of the paragraphs before and after do just that. In this way, the model description could be strengthened.

We thank the reviewer for the suggestion. To increase clarity, we now include several new figures (Figures 7, 8, 9, 10, 11) showing examples of low and high penalty grasps according to each penalty function.

M. Lines 392-3, "... can be applied to any set of novel objects ..." Yet this was not done. If the model is to be predictive, as described at various points in the MS, it needs to do just this.

We have included an additional experiment to address this issue, please see our response to previous reviewer comments.

N. Lines 423-439, Section "Neuroscience of Grasping" should be moved to the discussion or eliminated. This does not belong in the results section as there are no results here, nor in methods.

The Reviewer is mistaken. The section was always and remains in the Discussion section.

O. Methods, line 443. Can you be clear that there was no overlap between the subjects who were involved in Exps. 1 & 2?

Thanks for flagging this, we now specify this in the revised manuscript for all experiments (page 30 line 597):

"A different set of fourteen naïve participants (9 males and 5 females between the ages of 21 and 30, mean age: 24.4 years) participated in Experiment 2. An additional, different set of fourteen naïve participants (5 males and 9 females between the ages of 19 and 58, mean age: 25.1 years) participated in Experiment 3."

P. Methods, Apparatus, line 460. The Optotrak 3020 was used. This system (as I find in the on-line manuals) indicates a 0.1 mm 3d accuracy and 0.01 mm resolution. This seems quite good and suitable for your purposes. But it reads "... up to ..." Did you test what indeed the system could achieve? Is your separation between different measurements above these levels? It should at least be specified what are the device specs.

In our new analyses we show that model results are robust to mesh sampling distances up to 4 mm, well above the device specifications (page 24 line 450):

"Model performance is robust to relatively high levels of subsampling, and decreases only once sampled surface locations are on average more than 4 mm distant from one another (beyond 5% mesh subsampling)."

As suggested by the reviewer we now specify in the methods section of the revised manuscript that the device accuracy and resolution are sub-millimeter (page 31 line 615):

"We tracked participants' fingertip movements with sub-millimeter accuracy and resolution using an Optotrak 3020 infrared tracking system."

Q. Methods, lines 522. About 14% of trials were removed due to some form of error. Of those, more than 90% were due to tracking problems. Could it be that these are valid grasp strategies and common ones at that, but that the line of sight of the camera could not capture the necessary angles?

As we specify in the methods section of the manuscript (page 36 line 744):

"The trajectories of lost-tracking error trials, where the data are available, fall within the clusters of trajectories of corresponding non-error trials in 92.2% and 99.0% of cases across Experiments 1 and 2 respectively. In Experiment 3 the experimenter manually recorded grasp locations for error trials, and these locations are all represented in the final dataset. It is therefore unlikely that excluded error trials differed strongly from the data included in our analyses."

Reviewer #3: This is an elegant and new account of how humans choose grasping locations on 3D objects based on a simple normative theory. The theory is implemented by a computational model that combines five cost functions in order to locate on wooden block objects ideal precision grip locations. The model predicts quite well, even without free parameters, where humans place their index finger and thumb to firmly pick up these unfamiliar 3D objects.

I believe that the findings are new and interesting and that the manuscript may deserve to be published. I have, however, concerns that need to be addressed before the manuscript can be accepted for publication.

First, I wander how generalizable the model is beyond predicting grasp locations on "cube objects". Given the quite obvious assumptions of force closure and natural axis, the four objects tested do not offer a large number of alternative locations for stable grasps. Also, the constraint of limiting the grasps to 2.5cm, although based on previous studies, seems almost as it had been chosen after the empirical data collection, since subjects clearly preferred smaller grips. My point being, it looks like a quick inspection of the data about grip location selection presented in the supplementary material already shows evidence of what constraints humans use to select their grip, which makes the development of a computational model perhaps redundant.

We have added a section to the manuscript where we show model predictions on novel objects and find that these predictions well align with human grasps (page 23 line 420):

"**Model Predictions on Novel Objects.** The model was designed from the insights derived from Experiments 1 and 2 with polycube objects made of brass and wood. To test whether the model generalizes beyond this type of object, we selected four mesh models of objects with smooth, curved surfaces from an in-house database. We input these meshes to the model and generated grasp predictions (Figure 13a). The model was instantiated using the weights derived from Experiment 1. Next, we 3D printed these objects out of light plastic (~80g, comparable to Experiment 1 objects), and asked 14 human participants to grasp these novel objects. Figure 13b shows how human grasps agree with model predictions. Human and model grasps once again have similar size and orientation, and also cover similar portions of the objects. Figure 13c confirms this observation: predicted model grasps are as similar to medoid human grasps as grasps from a random human participant ($t(13)=1.53$, $p=0.15$)."

We also include perturbation analyses regarding the selected grip aperture threshold grasps (page 24 line 453):

"Since the backside of objects is occluded from view, it is unlikely that participants have an accurate estimate of grip aperture across the whole object. Additionally, since we constrained participants to two-digit precision grips, grasps above the threshold defined by Cesari and Newell (26) might be acceptable, as long as these are within a maximum comfortable grasp span. Figure 14b shows that indeed model performance is robust to increases in aperture threshold up to 100 mm."

Second, it seems to me that in Experiment 2 subjects pick-up the objects by basically ignoring the wooden parts, given how much heavier the brass parts are in comparison. Not surprisingly, the model does the same since the CoM is almost entirely determined by the brass parts. This is especially evident in the "near" and "far" conditions.

Agreed, demonstrating this behavior was indeed the objective of Experiment 2. Additionally, we now include an analysis, as suggested also by reviewers 1 and 2, showing how participants refine their preference for grasps close to the CoM throughout Experiment 2 (page 12 line 222):

"Figure ~~3g~~ 5a shows how human grasps are indeed significantly attracted towards the CoM of heavy objects, presumably to counteract the larger torques associated with higher mass. In Experiment 2, grasps were on average 11 mm closer to the object CoM than grasps sampled uniformly from the objects' surfaces (t(13)=4.94, p= 2.7*10-4), and on average 20 mm closer than the grasps from Experiment 1 (t(24)=6.63, p= 7.4*10-7). Figure 5b shows how this behavior was evident already from the very first trial performed by participants, but also that grasps clustered more toward the object CoM in later trials, presumably as participants refined their estimates of CoM location (correlation between CoM attraction and trial repetition: r = 0.86, p = 0.13). Importantly, participants shifted their grasps towards the CoM—not the geometrical centroid—of the objects (observe how the grasp patterns shift in Figure ~~3h~~5c)."

Third, the model is not based on an analysis of the 3D shape per se, since the input is a series of discrete grasping locations, which the model analyzes in a pair-wise fashion. If I understand the model correctly then I imagine there are many scrambled versions of the objects that would elicit the same predictions.

We don't fully understand what the reviewer means, so here are what we believe to be some pertinent observations. First, although each point in a penalty map refers to a unique combination of finger and thumb locations on the object's surface, the ordering of the axes of the cost functions is piecewise linear, such that neighboring points in the cost function refer to neighboring points on the object. Second, it's true that the penalty for each pair of locations is evaluated independently of the other points on the surface (no spatial derivatives, for example). Yet the values at each location do depend on the object shape as a whole (e.g., the magnitudes of torque, or visibility at a given surface point change as function of the rest of the object). So, the point about scrambling objects doesn't apply. Third, there is indeed a many-to-one mapping between object geometry and optimal grasp locations in allocentric space: multiple shapes can yield similar optimal grasps. But that is not a property of our model, just the fact that the space of possible objects is very large compared to the space of possible grasp poses. In all cases, if one were to drastically change the geometry of an object, the model would output different predictions, as we show throughout the manuscript. We have however now added a new section to the manuscript investigating model performance with different kinds of perturbations.

These analyses show that the model is robust to perturbations, including mesh subsampling (page 24 line 441):

"**Model Perturbation Analysis.** The model designed thus far receives as input a near-veridical representation of the objects to grasp. However, it is unlikely that humans have access to such a veridical object representation. We therefore implemented some perturbations to the inputs and key parameters of the computation model and observed how robust the model is to these perturbations. Specifically, we tested how model performance in predicting human grasping patterns from Experiment 3 varies as a functions of these perturbations.

The model input thus far consisted of densely sampled 3D mesh models. It's unlikely that humans also have such a dense, accurate 3D representation of an object's surface. Figure 14a therefore shows model performance (in terms of similarity with human grasping patterns) with different levels of surface mesh subsampling. Model performance is robust to relatively high levels of subsampling, and decreases only once sampled surface locations are on average more than 4 mm distant from one another (beyond 5% mesh subsampling)."

I summary, I think the model does a good job in predicting the empirical data. However, given the small set of objects tested and the reduced set of possible grasping locations that these objects offer, I am not sure what the model adds to what is already evident from a careful inspection of the raw data.

As mentioned above, we have now added an additional experiment demonstrating how the model is able to predict grasps on novel objects with different surface geometries than the ones employed in Experiments 1 and 2.
