# Supplementary material for: Predicting precision grip grasp locations on three-dimensional objects
Source: PLoS Comput Biol. 2020 Aug 4;16(8):e1008081. doi: 10.1371/journal.pcbi.1008081 (PMC7428291; doi:10.1371/journal.pcbi.1008081)
Supplement: Attachment — Submitted filename: Response_to_reviewers.pdf [file pcbi.1008081.s005.pdf]

We thank the reviewers for the positive assessment of our work. We address the remaining minor comments below.
Reviewer comments are written in italics. Our responses are in bold and inset. Changes to wording in the manuscript are highlighted in red text. Line and page numbers refer to the revised manuscript with tracked changes.

Reviewer #1: The authors have successfully addressed all the concerns I have raised in my previous review. I only have one additional minor comment:

The current implementation of the model is not considering that some parts of the object could act as obstacles. This would explain some of the patterns that can be observed in Figure S1(a). For example, when the S and V objects are in the S1, S2 and V1 orientations, there is one part of the object that is sticking up and thus obstructs possible grasps of the parts hidden behind it. Humans clearly take this aspect into account (they rarely moved the hand over the obstacle to grasp a part behind it). On the other hand, both the Unfitted and the Fitted models disregard the sticking part and choose grasps that differ from those made by humans. Interestingly, in S1(b) humans are now actually grasping over the obstacle because the cost of doing it is probably less than the cost of grasping the object to far away from the COM. The model could thus incorporate also a component that takes into account how long (or curved) the movements are. If a part of the object obstructs a direct reaching movement, the movement should be necessarily longer (more curved) and thus have a higher cost. I would say that at this stage is up to the authors to include this component into the model or just mention it as a possible limitation of the model.

> **This is an interesting observation. Indeed, it has been shown that the presence of obstacles can affect grasp selection (Voudouris, Smeets, & Brenner; 2012). However, this is likely due to the obstacles altering the configuration of the arm and hand during the grasp, since forcing participants to alter their reach-to-grasp trajectories does not on its own alter grasp selection (Voudouris, Brenner, Schot, and Smeets; 2010). We thank the reviewer for the comment and we now discuss in the revised manuscript how, in future work, obstacle avoidance might be included in the model (page 27 line 525):**
> "Relatedly, the effect of obstacles (and self-obstacles, such as the vertically protruding portions of some of the objects employed in this study) could also be assessed. The presence of obstacles could affect grasp selection by requiring reach-to-grasp trajectories that avoid an obstacle, although previous research has shown that forcing different hand paths does not affect selected grasp locations (25). Alternatively, the presence of obstacles might alter the configuration of the arm and hand during a grasp (43), which could be incorporated into the model by modifying the grip comfort penalties."

Reviewer #3: I am pleased with how the authors addressed my previous concerns. In particular, I am glad the model is able to generalize the predictions to novel objects that are substantially different from polycube objects made of brass and wood.

> **We thank the reviewer for their comments and we agree that showing how the model generalizes to novel objects was an important addition to the manuscript.**
